# Induced pain affects auricular and body biosignals: From cold stressor to deep breathing

**DOI:** 10.3389/fphys.2023.1090696

**Published:** 2023-01-17

**Authors:** Andrius Rapalis, Povilas Piartli, Lina Jankauskaitė, Vaidotas Marozas, Eugenijus Kaniusas

**Affiliations:** ^1^ Biomedical Engineering Institute, Kaunas University of Technology, Kaunas, Lithuania; ^2^ Department of Electronics Engineering, Faculty of Electrical and Electronics Engineering, Kaunas University of Technology, Kaunas, Lithuania; ^3^ Department of Pediatrics, Faculty of Medicine, Medical Academy, Lithuanian University of Health Sciences, Kaunas, Lithuania; ^4^ Institute of Physiology and Pharmacology, Medical Academy, Lithuanian University of Health Sciences Kauno Klinikos, Kaunas, Lithuania; ^5^ Institute of Biomedical Electronics, Faculty of Electrical Engineering and Information Technology, Vienna University of Technology (TU Wien), Vienna, Austria

**Keywords:** auricular bioimpedance, auricular vagus nerve stimulation, blood pressure, cold pressor test, deep breathing, physiological biofeedback, photoplethysmography

## Abstract

Pain affects every fifth adult worldwide and is a significant health problem. From a physiological perspective, pain is a protective reaction that restricts physical functions and causes responses in physiological systems. These responses are accessible for evaluation *via* recorded biosignals and can be favorably used as feedback in active pain therapy *via* auricular vagus nerve stimulation (aVNS). The aim of this study is to assess the significance of diverse parameters of biosignals with respect to their deflection from cold stressor to deep breathing and their suitability for use as biofeedback in aVNS stimulator. Seventy-eight volunteers participated in two cold pressors and one deep breathing test. Three targeted physiological parameters (*RR* interval of electrocardiogram, cardiac deflection magnitude *Z*_AC_ of ear impedance signal, and cardiac deflection magnitude *PPG*_AC_ of finger photoplethysmogram) and two reference parameters (systolic and diastolic blood pressures *BP*_S_ and *BP*_D_) were derived and monitored. The results show that the cold water decreases the medians of targeted parameters (by 5.6, 9.3%, and 8.0% of *RR*, *Z*_AC_, and *PPG*_AC_, respectively) and increases the medians of reference parameters (by 7.1% and 6.1% of *BP*_S_ and *BP*_D_, respectively), with opposite changes in deep breathing. Increasing pain level from relatively mild to moderate/strong with cold stressor varies the medians of targeted and reference parameters in the range from 0.5% to 6.0% (e.g., 2.9% for *RR*, *Z*_AC_ and 6.0% for *BP*_D_). The physiological footprints of painful cold stressor and relaxing deep breathing were shown for auricular and non-auricular biosignals. The investigated targeted parameters can be used as biofeedback to close the loop in aVNS to personalize the pain therapy and increase its compliance.

## 1 Introduction

Acute or chronic pain is one of the main complaints for seeking medical care. According to the International Association for the Study of Pain, pain is defined as “unpleasant sensory and emotional experience associated with, or resembling that associated with, actual or potential tissue damage” Raja et al. (2020). Pain is a protective reaction restricting physical functions with various physiological parameters, such as heart rate, respiratory rate, and arterial blood pressure, which are potential indicators of pain intensity Arbour et al. (2014); Cowen et al. (2015); Peters and Schmidt (1991). The specific changes can be observed by the reactivity and reflexivity of the autonomic nervous, cardiovascular, and respirator systems Kyle and McNeil (2014).

Adults and children suffer the pain associated with different medical conditions, undergo different painful procedures, or are referred with acute pain to the emergency department Dahlhamer et al. (2018); Keating and Smith (2011); Mura et al. (2017); Othow et al. (2022). Data suggest that 7 out of 10 patients come to the emergency department due to pain Todd et al. (2007). Meanwhile, chronic pain affects about 20.5% of adults in the United States Yong et al. (2022) and about 10%–30% in Europe Breivik et al. (2006); Reid et al. (2011). The meta-analysis showed that the prevalence of chronic pain ranges between 0% and 24% globally Mansfield et al. (2016).

Different medications, such as non-steroidal anti-inflammatory drugs, opioids, or others, are used daily against pain. Despite various pain medications and strategies, pain treatment faces many adversities Fishman (2007), such as severe side effects, the use of illicit drugs, opioid crisis St. Marie and Broglio (2020), peptic ulcers Tai and McAlindon (2021), and others. All the more, pain management is a fundamental human right Enright and Goucke (2016); Fishman (2007).

Vagal nerve stimulation (VNS)—as a pain neuromodulation technique, as reviewed in Kaniusas et al. (2019a)—has been investigated in humans and animals. VNS can affect the autonomic nervous system and is an approved treatment for pharmacoresistant depression and drug-resistant epilepsy Nemeroff et al. (2006); O’Reardon et al. (2006). Non-invasive transcutaneous modalities of VNS emerge Busch et al. (2013); Nesbitt et al. (2015), such as the electrical stimulation of the external surface of the ear innervated by the afferent auricular branch of the vagus nerve, known as auricular vagus nerve stimulation (aVNS). aVNS is performed using miniature electrodes tightly fixed inside the auricular concha. The current intensity is individually adjusted at the beginning of the aVNS session to a level without evoking pain. However, the initial personalization of the intensity of current alone does not ensure adequate vagus nerve stimulation for the relatively long treatment duration (from days to weeks). Here habituation effects, varying physiology, and deterioration of the electrode-tissue interface contribute to this uncertainty in the treatment Bouton (2017); Kaniusas (2019); Kaniusas et al. (2019b). Therefore, aVNS can be hypothesized to avoid under- or over-stimulation, reduce side effects, and save stimulation energy when based on individual physiological biofeedback.

Biofeedback can be assessed using data from internal (in-the-ear) and external (outside-the-ear) sensors, i.e., auricular and non-auricular biosignals. However, it is not known which biosignals and extracted parameters help estimate the balance between the stimulated parasympathetic system and the complementary sympathetic system, which is generally predominant in chronic ailments such as pain. In the ideal case, this balance should be provided to the aVNS stimulator to avoid the disadvantages of the non-personalized aVNS. Thus, easy-to-access biosignals are of high interest which could estimate this balance in favor of the efficiency of aVNS therapy.

The present study proposes a cold stressor as a sympathetically driven stimulus (usually accompanied by acute pain) and deep breathing as a mainly parasympathetically driven stimulus (with relaxing effects) to manipulate the sympathovagal balance from sympathetic to parasympathetic dominance while recording a set of auricular and non-auricular biosignals. The aim of this study is to assess the significance of diverse parameters of biosignals with respect to their deflection from cold stressor to deep breathing and their suitability for use as biofeedback in aVNS stimulator.

## 2 Materials and methods

### 2.1 Study population and data acquisition

Seventy-eight healthy volunteers (36 women), 32.6 ± 10.7 years old (range 20–64 years, with 23 men and 19 women < 30 years), with a height of 1.76 ± 0.1 m, a weight of 75.0 ± 13.6 kg, and a body mass index of 24.1 ± 3.7 kg/m^2^ participated in the study. All participants met the following criteria: 1) age ≥18 years; 2) no chronic pain; 3) no documented cardiovascular, respiratory, diabetes, and depression diseases; 4) no medication with b-blockers or calcium channel antagonists; and 5) no pregnancy or breastfeeding. Participants were instructed to avoid taking painkillers or anti-inflammatory drugs for at least 24 h and activities that could affect the cardiovascular system (smoking, coffee, alcohol, physical activity, medication, etc.) for at least 4 h before the study.

**FIGURE 1:**
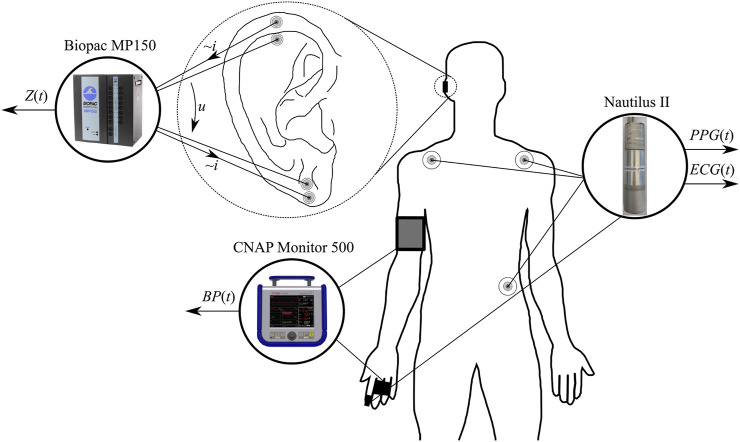
Placement of all sensors and electrodes for the recording of electrocardiogram *ECG*(*t*), pulse plethysmogram *PPG*(*t*), auricular impedance *Z*(*t*), and blood pressure *BP*(*t*) signals.

Data collection took place indoors at the Biomedical Engineering Institute (Kaunas, Lithuania) in a quiet and temperature-controlled (24.0°C ± 1.0°C) laboratory at the same time of the day (08:00–13:00) to minimize the circadian influence. Four synchronous biosignals were recorded in the study, as illustrated in Figure 1: 1) a modified bipolar three-lead electrocardiogram (ECG) signal (sampling rate 2 kHz); 2) a red wavelength finger photoplethysmogram (PPG) signal (sampling rate 1 kHz) using a proprietary multimodal signals recording system Nautilus II (Biomedical Engineering Institute, Kaunas, Lithuania); 3) an ear impedance signal (at the frequency of 12.5 kHz, sampling rate 1 kHz) using the data acquisition system Biopac MP150 (Biopac Systems Inc., Aero Camino, Goleta, CA, United States); and 4) arterial blood pressure signal (sampling rate 100 Hz) using the non-invasive arterial blood pressure monitoring system CNAP Monitor 500 (CNSystems, Graz, Austria). The subjective/perceived pain was recorded by a volunteer self-report (announced verbally and aloud to an experimenter) using the numerical rating scale NRS (range 0–100, with 0 for no pain and 100 for unbearable pain) at least every 30 s (or even more often based on a volunteer’s initiative).

**FIGURE 2:**
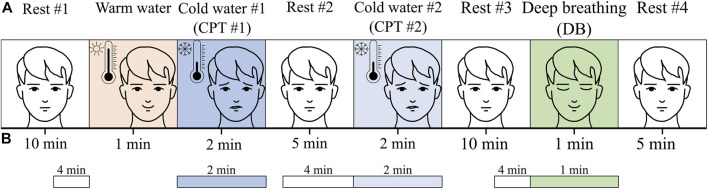
**(A)** The protocol of the study and **(B)** analyzed intervals.

Well-known and effective pain-causing (the cold pressor test) and relaxation (deep breathing) tests were used in the study. Namely, the study protocol consisted of eight phases, as depicted in Figure 2A: 1) the first rest phase (Rest #1) lasting 10 min in the sitting position; 2) the warm water for 1 min (equalization phase), in which the participant immersed his left hand into warm water (32.0°C ± 0.1°C); 3) the first cold water phase (CPT #1), in which the participant immersed his left hand into cold water (7.0°C ± 0.1°C) for 2 min or even shorter if the volunteer felt very uncomfortable and voluntarily resumed; 4) the second rest phase (Rest #2) for 5 min where the participant took his left hand out from cold water and rested in the sitting position; 5) the second cold water phase (CPT #2), in which the participant immersed his left hand into a little less cold water (10.0°C ± 0.1°C) for 2 min or even shorter if the volunteer felt very uncomfortable and voluntarily resumed; 6) the third rest phase (Rest #3) for 10 min in analogy with Rest #2; 7) the deep breathing phase (DB) for 1 min with the paced breathing rate 6 1/min (paced *via* a monitor and a bar rising/falling periodically every 10 s); 8) the fourth rest phase (Rest #4) for 5 min in analogy with Rest #2. Participants were verbally instructed to immerse their left hand (up to the middle of the forearm) in warm or cold water, indicate their subjective pain level (in cold water), and take out their hand after 1 min in warm and 2 min in cold water.

The study was conducted following the ethical principles of the Declaration of Helsinki and with ethics approval from the Kaunas Region Biomedical Research Ethics Committee (No. BE-2-24), including informed consent and voluntary participation. Personal information was removed from the collected data to ensure participants’ anonymity.

### 2.2 Signal processing and parameters extraction

**FIGURE 3:**
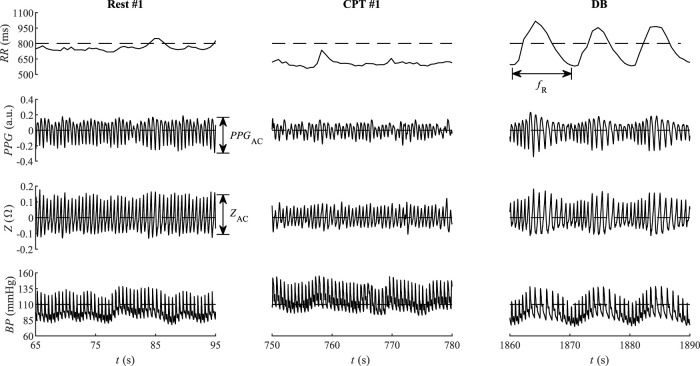
Instructive qualitative changes of the interbeat period *RR*, the pulse plethysmography *PPG*, bioimpedance *Z*, and blood pressure *BP* from the first rest phase (Rest #1), the first cold water phase (CPT #1), and the deep breathing phase (DB) of a single participant. The maximum reported NRS value of the CPT #1 was 80.

ECG was filtered using zero-phase Butterworth high-pass and low-pass filters (cut-off frequencies 0.5 and 35 Hz, respectively), R waves were detected using the modified Tompkins algorithm Hamilton and Tompkins (1986), and *RR* was estimated as the time interval between the successive R peaks. The ear impedance signal reflects local changes in the blood perfusion and blood vessel size, accounting for the local changes in capacitance and resistance. The impedance signal is morphologically similar to PPG so that both *PPG* and *Z* were filtered using high-pass and low-pass zero-phase Butterworth filters (cut-off frequencies 0.5 and 10 Hz, respectively). The associated peak and valley fiducial points in *PPG*, *Z*, and *BP* signals were detected in line with the detected R waves of ECG. Five parameters were extracted out of the four recorded biosignals (Figure 3): 1) time interval *RR* between R peaks of ECG; 2) cardiac deflection magnitude *PPG*_AC_ of PPG; 3) cardiac deflection magnitude *Z*_AC_ of ear impedance signal; 4) systolic blood pressure *BP*_S_; and 5) diastolic blood pressure *BP*_D_. Please note that the analyzed *PPG*_AC_ is mainly related to the pulsatile arterial blood, proportional to the local systolic-diastolic deflection of the blood pressure and the arterial compliance of the vascular wall Kaniusas (2015).

The entire periods of stimulation phases CPT #1, CPT #2, and DB were included in the analysis, only the last 4 min of rest phases Rest #1, Rest #2, and Rest #3 were included to avoid the transient influence of the preceding phase (Figure 2B). The medians of evaluated parameters from CPT #1, CPT #2, and DB phases were compared with the medians of the respective Rest #1, Rest #2, and Rest #3 phases, without any averaging. The analysis was performed using different pain levels, ages, and gender. The pain level threshold was chosen at 40 points, corresponding to mild pain Karcioglu et al. (2018). The age threshold was selected at 30 years in order to end up with comparably populated groups of men and women.

### 2.3 Statistical analysis

The Shapiro-Wilk test was used to assess data normality. Because of the non-normal distribution, the results are summarized using boxplots with medians and quartiles. The Wilcoxon signed-rank test with the Bonferroni’s adjustment for dependent samples was used to compute the *p*-value, and statistical significance was set at *p* < 0.05.

## 3 Results

Out of 78 recorded data sets, two *ECG*, thirteen *PPG*, twenty-four *Z*, and six *BP* traces were eliminated from the analysis due to poor quality. Eight participants retreated earlier and did not finish the CPT #1 phase, i.e., two women (age ≤30 years), two women (age > 30 years), one man (age ≤30 years), and one man (age > 30 years). Two other participants did not finish CPT #2, i.e., one woman and one man, both aged > 30 years.

### 3.1 General tendencies

Figure 3 illustrates the temporal courses of *RR*, *PPG*, *Z*, and *BP* during phases Rest #1, CPT #1, and DB. Compared to Rest #1, the cold stimulus CPT #1 shows reduced both *RR* and its variability, as well as reduced cardiac deflection magnitude *PPG*_AC_ of *PPG* and reduced cardiac deflection *Z*_AC_ of *Z*. The associated mean *BP* is larger during CPT #1 than during Rest #1. The subsequent DB phase contrasts CPT #1 in that *PPG*_AC_ and *Z*_AC_ increase in DB. The respiration-related variability of all four *RR*, *PPG*_AC_, *Z*_AC_, and *BP* dominates in DB, with the indicated respiration rate *f*_R_ (Figure 3).

### 3.2 Cold water versus deep breathing

**FIGURE 4:**
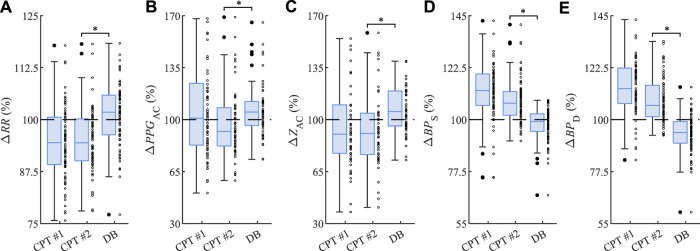
Relative changes **(A)** Δ*RR* of *RR*, **(B)** Δ*PPG*_AC_ of *PPG*_AC_, **(C)** Δ*Z*_AC_ of *Z*_AC_, **(D)** Δ*BP*_S_ of systolic BP values, and **(E)** Δ*BP*_D_ of diastolic BP values from CPT #1, CPT #2, and DB as related to the respective Rest #1, Rest #2, and Rest #3. The asterisk ”*” indicates significant changes (*p* < 0.05) between CPT #2 and DB.

As shown in Figure 4, the first cold water stimulation CPT #1 decreases the median of *RR* (−5.5%) and *Z*_AC_ (−9.8%) while increasing that of *BP*_S_ (+12.6%) and *BP*_D_ (+13.4%) of BP, as compared with the first rest phase Rest #1. Here the associated *PPG*_AC_ remains almost constant (+0.9%). The second cold water stimulation CPT #2 decreases the median of *RR* (−5.6%), *PPG*_AC_ (−8.0%), and *Z*_AC_ (−9.3%) while increasing that of *BP*_S_ (+7.1%) and *BP*_D_ (+6.1%), as compared with the second rest phase Rest #2. The subsequent deep breathing DB produces opposite effects: the median of *RR* (+1.8%), *PPG*_AC_ (+5.1%), and *Z*_AC_ (+5.4%) increase, while that of *BP*_S_ (−0.9%) and *BP*_D_ (−5.6%) decrease, as compared with the third rest phase Rest #3. The observed changes in DB are significantly different compared to CPT #2 in all five parameters.

**FIGURE 5:**
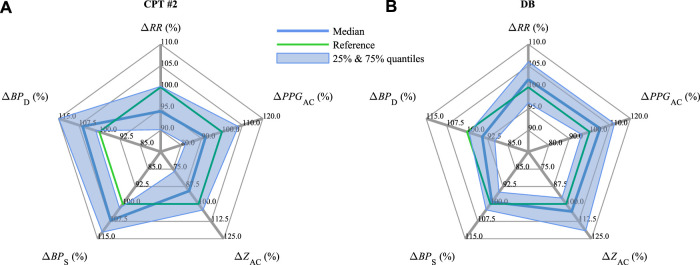
Medians and interquartile ranges of Δ*RR*, Δ*PPG*_AC_, Δ*Z*_AC_, Δ*BP*_S_, and Δ*BP*_D_ (compare Figure 4) during **(A)** CPT #2 and **(B)** DB.

Figure 5 summarizes and contrasts the observed changes for all parameters in CPT #2 (Figure 5A) versus DB (Figure 5B), with the indicated interquartile range from 25% to 75%. In line with Figure 4, CPT #2 reduces *RR*, *PPG*_AC_, and *Z*_AC_ and increases *BP*_S_ and *BP*_D_, whereas DB causes physiological processes with reversed tendencies, i.e., *RR*, *PPG*_AC_, and *Z*_AC_ increase while *BP*_S_ and *BP*_D_ decrease.

### 3.3 Pain level differences

**FIGURE 6:**
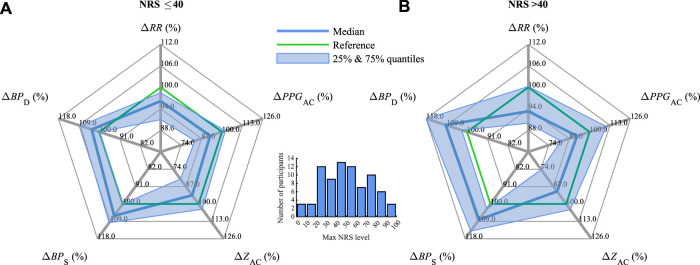
Median and interquartile ranges of Δ*RR*, Δ*PPG*_AC_, Δ*Z*_AC_, Δ*BP*_S_, and Δ*BP*_D_ (compare Figure 4) during CPT #2 for **(A)** mild pain with NRS ≤40 and **(B)** moderate to severe pain with NRS > 40. The distribution of maximum self-report NRS of the CPT #2 phase is presented in a bar diagram.

Figure 6 illustrates the relative changes in the parameters in CPT #2 for relatively mild pain with the associated NRS ≤40 (Figure 6A) in comparison with moderate to strong pain with NRS > 40 (Figure 6B). It can be observed that the physiological changes for NRS ≤40 are more closely located to the 100% reference line, i.e., to the values in Rest #2, than for NRS > 40. Namely, the median Δ*RR* decreases by −3.8% and −6.7% for NRS ≤40 and NRS > 40, respectively; the associated Δ*PPG*_AC_ decreases by −7.5% and −8.0%, Δ*Z*_AC_ decreases by −6.5% and −9.4%, Δ*BP*_S_ increases by +6.1% and +8.1%, and Δ*BP*_D_ increases by +3.4% and +9.4%. When comparing NRS ≤40 and NRS > 40, statistically significant changes are observed in *BP*_D_ only.

### 3.4 Gender and age tendencies

**FIGURE 7:**
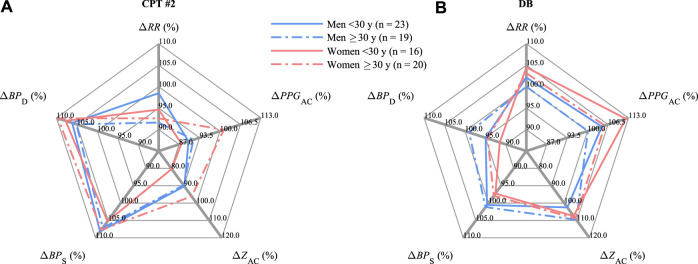
Medians of Δ*RR*, Δ*PPG*_AC_, Δ*Z*_AC_, Δ*BP*_S_, and Δ*BP*_D_ (compare Figure 4) differentiated by age and gender during **(A)** CPT #2 and **(B)** DB.

The influence of gender and age is depicted in Figure 7 considering CPT #2 and DB (compare Figure 5). In CPT #2, the relative values of Δ*RR* decrease by 1.5%–8.4%, with a minor decrease for young men ( < 30 years) and the largest decrease for adult men (≥30 years). Here Δ*PPG*_AC_ decreases by 8.4%–12.3%, with almost no changes for adult women (≥30 years). Δ*Z*_AC_ decreases by 3.9%–19.7%, with little changes for adult women and maximum changes for young women ( < 30 years). Δ*BP*_S_ increases by 5.3%–8.2% with minor changes for young women, whereas Δ*BP*_D_ increases by 5.1%–9.7%, with the largest changes for adult women.

During DB, in line with Figure 5, the observed changes in all gender and age groups mainly follow the opposite behavior. Δ*RR* increases by 2.1%–4.5% except for adult men (≥30 years); Δ*PPG*_AC_ increases by 4.5%–12.1% but also except for adult men; Δ*Z*_AC_ increases by 2.5%–9.6% with a minor increase for young men ( < 30 years); Δ*BP*_S_ increases very slightly for men (0.5%–1.1%) but decreases more strongly for women (2.1%–2.8%); Δ*BP*_D_ decreases in all cases by 1.0%–8.5% with a minor decrease for adult men (≥30 years) and the largest decrease for young women ( < 30 years).

## 4 Discussion

The present study investigates the physiological footprints of auricular and non-auricular biosignals in response to a cold stressor and deep breathing. While a cold stressor is a sympathetically driven stimulus (accompanied by acute pain), deep breathing is a mainly parasympathetically driven stimulus (with relaxing effects). Thus, it was investigated how the opposing sympathetic and parasympathetic stimuli are reflected by the auricular biosignals, namely, its parameter *Z*_AC_, and by parameters accessible from the auricular biosignals such as *RR* and *PPG*_AC_. All these three parameters can be used as biofeedback to close the loop in aVNS, i.e., in a targeted stimulation of the parasympathetic system. The closed-loop set-up personalizes aVNS with an expected tendency to avoid over and under-stimulation of the vagus nerve/parasympathetic system. Thus, the closed-loop aVNS may minimize both the energy consumption of the aVNS stimulator and potential side effects (no over-stimulation) while optimizing and personalizing the aVNS therapy (no recruitment of pain fibers), e.g., in chronic pain. Here, the non-auricular biosignals with their parameters *BP*_S_ and *BP*_D_ serve as a necessary reference to monitor stimuli-related vital functions of the body and as an instructive substrate for their comparison with stimuli-related changes in auricular biosignals.

The auricular biosignal *Z*, namely its parameter *Z*_AC_ (Figure 1), decreases significantly during the sympathetic stimulus (CPT #2) as compared with the parasympathetic one (DB) (Figures 3–5), as well as decreases tendentially with increasing pain perception (Figure 6). This behavior indicates the potential suitability of *Z*_AC_ in assessing changes in the balance of the parasympathetic and sympathetic stimuli, or, more generally, in the balance of the parasympathetic and sympathetic systems of the human body (sympathovagal balance). On the other hand, this balance, especially its normalization from a derailed state, is usually a therapeutic target in aVNS when applied to different chronic ailments Kaniusas et al. (2019a). Thus *Z*_AC_ can be hypothesized to be reasonable auricular biofeedback for the closed-loop aVNS without using any sensors external to the ear, which may obstruct the patient.

The parameters *RR* and *PPG*_AC_ also reflect sympathovagal balance. *RR* and *PPG*_AC_ decrease significantly during the sympathetic CPT #2 compared to the parasympathetic DB (Figures 3–5). While *RR* tends to decrease with increasing pain, the level of *PPG*_AC_ does not (Figure 6). Therefore, *RR* and *PPG*_AC_, the former to a larger extent, can also be hypothesized to be reasonable auricular biofeedback for the closed-loop aVNS targeting a derailed sympathovagal balance. Please note that *RR* could be estimated from the period of the cardiac oscillation of the auricular *Z* (Figure 3), whereas *PPG*_AC_ from the cardiac deflection of *PPG* from the earlobe Allen (2007). However, limitations in the precision of the estimated *RR* may apply in the former case due to a rather smooth waveform of *Z* in contrast to the spiky R peak of *ECG*. Likewise, limitations in *PPG*_AC_ may apply in the latter case due to a rather central connection of the ear perfusion in contrast to the peripheral perfusion of the finger (Figure 1).

The non-auricular parameters *BP*_S_ and *BP*_D_ reflect the sympathovagal balance as well. Both increase significantly during the sympathetic CPT #2 as compared with the parasympathetic DB (Figures 3–5), while this increase in CPT #2 tends to be larger for stronger pain (Figure 6). The level of *BP*_D_ appears to depend even stronger on the stimuli-induced sympathovagal balance with the observed changes of 11.7% (from CPT #2 to DB) in contrast to the associated changes in *BP*_S_ of 8.0% (Figure 4). Likewise, the sympathetically-governed vasoconstriction (governing *BP*_D_) may be more dominant than stroke volume changes (governing *BP*_S_) Kaniusas (2012). This leads to a hypothesis that *BP*_S_ and *BP*_D_ could be used as non-auricular biofeedback for the closed-loop aVNS when external sensors are used outside the ear.

In terms of gender and age, the largest changes from CPT #2 to DB were shown in *RR* for adult women (≥30 years), *PPG*_AC_ for young women ( < 30 years), *Z*_AC_ for young women ( < 30 years), *BP*_S_ for adult women (≥30 years), *BP*_D_ for young women ( < 30 years). In contrast, the minor changes from CPT #2 to DB were shown in *RR* for young men ( < 30 years), *PPG*_AC_ for adult men (≥30 years), *Z*_AC_ for young men ( < 30 years), *BP*_S_ for adult men (≥30 years), *BP*_D_ for adult men (≥30 years). Overall, men seem to show fewer changes from CPT #2 to DB than women. This conclusion is in line with previous studies, which conclude that women are more sensitive to pain Fillingim et al. (2009); Mogil (2012); Popescu et al. (2010), but it depends on the method of pain induction and assessment. In most cases, the study also supports the still controversial claims that older individuals are more tolerant of pain and show fewer physiological effects than younger individuals Edwards et al. (2003); Riley et al. (2010); Rittger et al. (2011). However, these statements are very limited in their validity due to the small sample in this study.

A limitation of the present study is the relatively small database of recordings representing the elder part of the population which has tendentially a larger prevalence of suffering pain. Collecting and analyzing a more representative database is planned as a future research direction in the research of the aVNS stimulator. Since the warm water phase immediately preceding CPT #1 strongly affected the results in CPT #1, we focused our investigations on the comparison of CPR #2 and DB, both preceded by rest phases. Another limitation is that the order of the different phases of the protocol were not randomized, especially the order of CPT and DB. Therefore, the results may have been influenced by other factors such as expectation, adaptation, prolonged exposure.

Lastly, it should be noted that the recorded pain level, in contrast to nociception with its physiological encoding and processing of nociceptive stimuli, is a subjective feeling connected with the emotional experience to impeding or actual harm Loeser and Treede (2008) but also altering autonomic nervous system Woo et al. (2017); Adamczyk et al. (2020); Abdallah and Geha (2017). Thus, the investigated objective characteristics of the autonomic system may be useful for a continuous and objective personalization of aVNS in chronic ailments such as pain.

## 5 Conclusion

The three parameters *RR*, *PPG*_AC_, and *Z*_AC_ accessible from auricular biosignals reflect the artificially-induced stimuli with sympathetic or parasympathetic dominance and thus the sympathovagal balance derailed in pain and other chronic conditions. Therefore, auricular biosignals can be used as biofeedback to close the loop in auricular vagus nerve stimulation to personalize the strength and timing of the stimulation in favor of therapy, patient compliance, and resourceful energy use.

## Data Availability

The raw data supporting the conclusions of this article will be made available by the authors, without undue reservation.
